# Recommendations for the classification of germline variants in the exonuclease domain of POLE and POLD1

**DOI:** 10.1186/s13073-023-01234-y

**Published:** 2023-10-17

**Authors:** Pilar Mur, Julen Viana-Errasti, Sandra García-Mulero, Lorena Magraner-Pardo, Inés G. Muñoz, Tirso Pons, Gabriel Capellá, Marta Pineda, Lidia Feliubadaló, Laura Valle

**Affiliations:** 1 Hereditary Cancer Program, Catalan Institute of Oncology, IDIBELL, Hospitalet de Llobregat, Barcelona, Spain; 2 Program in Molecular Mechanisms and Experimental Therapy in Oncology (Oncobell), IDIBELL, Hospitalet de Llobregat, Barcelona, Spain; 3 Centro de Investigación Biomédica en Red de Cáncer (CIBERONC), Madrid, Spain; 4 Department of Health of Catalonia, Catalan Cancer Plan, Barcelona, Spain; 5 Unit of Biomarkers and Susceptibility, Oncology Data Analytics Program (ODAP), Catalan Institute of Oncology, Hospitalet de Llobregat, Barcelona, Spain; 6 The CRUK Gene Function Laboratory and The Breast Cancer Now Toby Robins Research Centre, The Institute of Cancer Research (ICR), London, UK; 7 Protein Crystallography Unit, Structural Biology Program, Spanish National Cancer Research Center (CNIO), Madrid, Spain; 8 Department of Immunology and Oncology, National Center for Biotechnology (CNB-CSIC), Spanish National Research Council, Madrid, Spain

**Keywords:** Polymerase proofreading-associated polyposis, PPAP, Polymerase epsilon, Polymerase delta, Proofreading deficiency, Mutational signatures, Variant classification, Hereditary cancer

## Abstract

**Background:**

Germline variants affecting the proofreading activity of polymerases epsilon and delta cause a hereditary cancer and adenomatous polyposis syndrome characterized by tumors with a high mutational burden and a specific mutational spectrum. In addition to the implementation of multiple pieces of evidence for the classification of gene variants, *POLE* and *POLD1* variant classification is particularly challenging given that non-disruptive variants affecting the proofreading activity of the corresponding polymerase are the ones associated with cancer. In response to an evident need in the field, we have developed gene-specific variant classification recommendations, based on the ACMG/AMP (American College of Medical Genetics and Genomics/Association for Molecular Pathology) criteria, for the assessment of non-disruptive variants located in the sequence coding for the exonuclease domain of the polymerases.

**Methods:**

A training set of 23 variants considered pathogenic or benign was used to define the usability and strength of the ACMG/AMP criteria. Population frequencies, computational predictions, co-segregation data, phenotypic and tumor data, and functional results, among other features, were considered.

**Results:**

Gene-specific variant classification recommendations for non-disruptive variants located in the exonuclease domain of *POLE* and *POLD1* were defined. The resulting recommendations were applied to 128 exonuclease domain variants reported in the literature and/or public databases. A total of 17 variants were classified as pathogenic or likely pathogenic, and 17 as benign or likely benign.

**Conclusions:**

Our recommendations, with room for improvement in the coming years as more information become available on carrier families, tumor molecular characteristics and functional assays, are intended to serve the clinical and scientific communities and help improve diagnostic performance, avoiding variant misclassifications.

**Supplementary Information:**

The online version contains supplementary material available at 10.1186/s13073-023-01234-y.

## Background

The major function of polymerases is to replicate the genome, which is performed by polymerases, α, ε and δ in eucaryotes. Unlike α, polymerases ε (Polε) and δ (Polδ) contain an active 3'-5' exonuclease domain (ED) which proofreads newly synthesized DNA for replication errors. Polε and Polδ are comprised of four subunits in humans, the largest of which contains the catalytic polymerase and exonuclease domains and is encoded by the genes *POLE* and *POLD1 *respectively [1, 2].

Heterozygous germline pathogenic variants affecting the proofreading activity of Polε and Polδ cause increased risk to develop adenomatous polyposis and colorectal cancer (CRC), as well as endometrial, ovarian, breast, brain and upper gastrointestinal cancers, among other tumors [3–6]. This autosomal dominant cancer syndrome is called polymerase proofreading-associated polyposis (PPAP; MIM# 615083, 612591). The associated clinical features are usually developed in the adult age, except for rare aggressive cases that present with a constitutional mismatch repair deficiency (CMMRD)-like phenotype in childhood or adolescence [7–9]. Somatic *POLE *ED pathogenic variants occur in 7–15% of endometrial cancers [10–13], 0.5–8% of colorectal tumors [14–17], and more rarely in brain tumors (gliomas), extracolonic gastrointestinal cancers, and other tumor types. Somatic *POLD1* ED mutations are extremely rare.

As mentioned above, the ED determines the proofreading function of Polε and Polδ, which is essential for replication fidelity. Therefore, Polε and Polδ exonuclease disruption by pathogenic variants, either germline or somatic, leads to the accumulation of thousands of variants in the tumors (> 10 somatic variants per Mb (mut/Mb), and often, > 100) [12, 18–20]. Moreover, they present a characteristic variant spectrum, enriched in C > A transversions in the context of TCT, and C > T transitions in the context TCG [15, 21], which corresponds to tumor mutational signatures SBS10a, SBS10b, and SBS28 [22] for Polε proofreading defects, and SBS10d and SBS10c (identified in unaffected tissues) for Polδ proofreading deficiency, of the Catalogue Of Somatic Mutations In Cancer (COSMIC) (https://cancer.sanger.ac.uk/signatures/sbs/Mutational Signatures v3.2) [21, 23]. Occasionally, polymerase proofreading deficiency co-occurs with DNA mismatch repair (MMR) deficiency (dMMR) in the tumors. In that scenario, the tumor mutational signatures present are SBS14 (Polε proofreading deficiency + dMMR), and SBS20 (Polδ proofreading deficiency + dMMR) [15, 21, 24]. Hereditary and sporadic proofreading-deficient tumors, due to the strong immunogenicity elicited by the high mutation rate (strong neoantigen expression), show favorable prognosis and clinical benefit from immune checkpoint blockade [25–30].

Constitutional loss-of-function variants and variants located outside the exonuclease domain of *POLE* and *POLD1* do not cause the cancer predisposition syndrome PPAP; however, they may predispose to autosomal recessive or dominant congenital disorders. FILS syndrome (MIM# 615139), a very rare recessive Mendelian disorder characterized by facial dysmorphism, immunodeficiency, livedo, short stature, and variable skin manifestations, is caused by *POLE *pathogenic variants located outside the exonuclease domain and/or disrupting the encoded protein [31–33]. Biallelic *POLE *pathogenic variants have also been associated with another rare Mendelian syndrome, IMAGE-I (MIM# 618336), characterized by intrauterine growth retardation, metaphyseal dysplasia, adrenal hypoplasia congenita, genital anomalies, immunodeficiency, and diffuse large B-cell lymphoma [34, 35]. None of the patients tested with the congenital disorders herein mentioned show complete lack of *POLE* expression, suggesting that this would be lethal to the embryo. Constitutional heterozygous *POLD1 *pathogenic variants that impair the polymerase (replicative) activity of Polδ (dominant negative effect), cause an autosomal dominant progeroid syndrome called MDPL (MIM# 615381), characterized by mandibular hypoplasia, deafness, progeroid features, and lipodystrophy [36, 37].

Accurate *POLE* and *POLD1* ED variant classification, which is the focus of this article, is of utmost importance due to the consequences for the correct clinical management of ED variant heterozygotes and their families, impacting clinical surveillance based on specific cancer risks, as well decision making in oncology, based on the predictive value of ED mutations for prognosis and response to immunotherapy.

The American College of Medical Genetics and Genomics and the Association for Molecular Pathology (ACMG/AMP) developed generic variant classification guidelines that include criteria with varying levels of strength for and against pathogenicity, based on evidence gathered from multiple sources, including population data, computational and predictive data, phenotype/family history information, and functional data [38]. These recommendations allow the classification of variants into five categories: pathogenic (P), likely pathogenic (LP), variant of uncertain significance (VUS), likely benign (LB), and benign (B). Despite their value, these guidelines are generic for any Mendelian disease-causative gene and do not take into consideration gene and/or syndrome-specific particularities. Here we present specific recommendations to apply the ACMG/AMP guidelines for the classification of variants located in the ED of *POLE* and *POLD1*, -where the variants associated with cancer predisposition are found-, and the scientific rationale applied for their definition. We also present the curated classification of 128 ED missense variants after applying the recommendations that we propose. These recommendations have been developed to be made available to the scientific and clinical communities until official recommendations from the InSiGHT/ClinGen Hereditary Colorectal Cancer and Polyposis Variant Curation Expert Panel (https://clinicalgenome.org/affiliation/50099/) are published.

## Methods

### ACMG/AMP variant classification guidelines

Assessment of each ACMG/AMP rule code and evaluation of their utility for the classification of *POLE* and *POLD1* ED variants was performed. Previously published specifications developed by ClinGen Variant Curation Expert Panels (VCEP) were taken into consideration (https://cspec.genome.network/cspec/ui/svi/summary), in particular those defined for cancer predisposition genes [39–43].

ACMG/AMP rules were divided into four types of evidence: (i) population data; (ii) variant nature, variant location and computational predictive data; (iii) segregation and phenotypic data, including tumor mutational data; and (iv) functional data. As per ACMG/AMP guidelines, evidence in each category have varying levels of strength: very strong (PVS), strong (PS), moderate (PM), and supporting (PP) for pathogenic criteria; and stand-alone (BA), strong (BS), and supporting (BP) for benign criteria. All 28 original criteria were evaluated for their application to *POLE* and *POLD1* ED variant classification. Rule codes that were irrelevant to *POLE* and *POLD1 *or the syndrome, or for which limited data was available, or that included redundant information with another criterion, or that had been removed by the ClinGen Sequence Variant Interpretation (SVI) working group [44], were excluded. Criteria modifications included gene- or disease-specific modifications, strength-level adjustments, general recommendations, and certain criteria deemed not applicable.

For the final variant classification, recommendations provided in the manuscript have been followed, and the standard ACMG/AMP combination rules to define pathogenic, likely pathogenic, likely benign and benign variants were applied (Additional file 1: Table S1) [38].

PM and LV performed the classification of the 128 variants in parallel, without access to the other researcher’s classification. Complete concordance between the two classifications was reached for all variants.

### Variant nomenclature

Variant nomenclature follows HGVS recommendations (v.20.05), with nucleotide 1 corresponding to the A of the ATG translation initiation codon. All variants were annotated according to RefSeq IDs LRG_789; NM_006231.4 (*POLE*) and LRG_785; NM_001256849.1 (≈NM_002691.4) (*POLD1*). POLE ED includes amino acids 268–471, and POLD1 ED, amino acids 304–533 (based on NCBI: “region_name DNA_polB_epsilon_exo and DNA_polB_delta_exo").

### Population data

The Genome Aggregation Database [45] (gnomAD, non-cancer dataset; https://gnomad.broadinstitute.org/) was used as source of publicly available population control data (as of today, gnomAD non-cancer v.2.1.1, as it is the largest available dataset: 134,187 individuals, 50,913 of whom are non-Finnish Europeans), ignoring the frequencies observed in populations with high potential for founder effects, such as Ashkenazi Jewish or Finnish sub-populations, and the unclear ancestry “Population: other”.

### In silico predictions

In silico predictions of pathogenicity were performed with SIFT [46], PolyPhen-2 [47], CADD [48, 49] and the metapredictor REVEL [50], which combines pathogenicity predictions and conservation information obtained from 18 individual scores. Scores were obtained from the Variant Effect Predictor (VEP) web tool [51]. The BLOSUM62 matrix was used to score pairs of aligned residues [52].

### 3D modeling: DNA binding cleft

3D models based on the crystallographic structure of the homologous yeast proteins Pol2 (PBD ID: 4m8o) and Pol3 (PDB ID: 3iay, chain A), with a single-stranded DNA (ssDNA) from the aligned bacteriophage T4 polymerase complex (PDB ID: 1noy) located in the proper position for exonuclease proofreading, were used to evaluate the location of the affected amino acids in the 3D structure of POLE and POLD1. Structural superpositions, refinement, and manual adjustments to the 3D models of human POLE and POLD1 in complex with ssDNA were performed with COOT [53].

The DNA binding cavity was defined according to CASTp (http://sts.bioe.uic.edu/castp/calculation.html). Interatomic distances were calculated with ContPro (http://procarb.org/contpro/). Direct contact of an amino acid with the ssDNA (positioned for proofreading) was defined when any atom of the amino acid is accessible to the cavity where the DNA binds and at less than 6 Å from the ssDNA. Indirect contact is defined when any atom of the amino acid is accessible to the cavity but at ≥ 6 Å from the ssDNA. No contact was considered when the atoms of an amino acid are, in the 3D models, at > 6 Å from the ssDNA (ContPro) and not accessible to the DNA-binding cavity (CASTp). Additional file 2: Table S2 indicates the predictions for each residue in the ED of POLE and POLD1.

### Tumor mutational burden and signatures

Exome or genome sequencing data processing for the calculation of tumor mutational burden and COSMIC mutational signatures was performed as previously specified [5]. Total mutation burden was estimated by considering single nucleotide variants (SNV) from exonic regions and with a variant allele frequency higher than 10%. The number of mutations per megabase (mut/Mb) was calculated as the total mutational burden divided by the genomic exome length (32.95 Mb). The contribution of tumor mutational signatures was calculated with FitMS through the Signal web application (https://signal.mutationalsignatures.com/), not selecting tissue-specific signatures (access date: November 2022). In Signal, COSMIC v.3 signatures were considered when evaluating a *POLE* variant, since they include, among others, SBS10a, SBS10b, SBS28, SBS14 and SBS20. For *POLD1* variants, Cancer Reference Signatures (CRS) were considered, which include, among others, SBS10a, SBS10d, SBS14 and SBS20.

Exome sequencing data (BAM files) or targeted sequencing data (≥ 100 genes analyzed) from tumors harboring the *POLE* and *POLD1* ED variants identified in inherited cases were obtained from TCGA (accessed May 2021) and/or COSMIC v.94 (accessed May 2021).

### Analysis of the specificity of mutational signatures associated with proofreading deficiency

Two subgroups of samples, obtained from TCGA, were considered based on the ED mutational status: 68 proofreading deficient TCGA tumor samples, and 70 without mutations in the exonuclease domain of *POLE* or *POLD1*, randomly selected (gastric, colorectal, and endometrial cancers). Sequencing data processing was performed as described above.

The clustering of the samples was performed based on the percentages of contribution of polymerase proofreading deficient-associated signatures: SBS10a, SBS10b, SBS10d, SBS28, SBS14 and SBS20 [23, 54]. The distances among samples were computed via R function dist, with Euclidean distance. Subsequently, hclust function was used to generate the clustering based on the distances calculated with the Ward-D2 linkage method. For visualization purposes, data was plotted in a heatmap using the ComplexHeatmap package.

### Tumor MMR deficiency

Tumor MMR status was obtained from the data reported in TCGA, whenever available. MMR deficiency (microsatellite instability, MSI) was established in TCGA based on the estimations retrieved from MANTIS [55] (cutoff: 0.4) and MSIsensor [56] (cutoff: 3.5). When the MSI Bethesda panel [57, 58] results were available, this status was prioritized. Only one sample showed discordant results between the MSI panel and the TCGA determinations.

## Results and discussion

### Training set of pathogenic and benign ED variants to define the usability and strength of the ACMG/AMP criteria

Literature and database searches were performed using PubMed, Mastermind, gnomAD, and ClinVar (accessed February 2021), and variants with strong evidence of pathogenicity or benignity/neutrality were considered to define a training set of variants used to help in the definition of the specifications of the ACMG/AMP guidelines. Variant selection was based on a simplistic model where strong pieces of evidence in favor of pathogenicity or neutrality were considered (Table 1): 17 variants (13 in *POLE* and 4 in *POLD1*) were considered pathogenic, and 5 (3 in *POLE* and 2 in *POLD1*) benign (Table 2; Additional file 3: Table S3).

**Table 1:** Evidence scoring system to select the pathogenic and benign ED variants that were used to define the usability and strength of the ACMG/AMP criteria

In favor of pathogenicity	In favor of neutrality
MAF < 0.0005% (all gnomAD v.2.1.1 non-cancer populations) *AND:* Somatic hotspot (≥ 10 tumors) *OR* Recurrent in PPAP families (≥ 3 families) and evidence of cosegregation with PPAP tumors^b^ in at least one family *OR* Variant affects a catalytic exonuclease site, and the residue change translates into a negative BLOSUM62 score *OR* Proofreading defective-associated mutational signatures SBS10, SBS14 and/or SBS20 identified in ≥ 2 tumors *OR* Variant is identified in a patient with a CMMRD-like phenotype in the absence of CMMRD (absence of germline biallelic MMR gene mutations) *OR* De novo germline variant in a patient with a tumor harboring SBS10, SBS14 and/or SBS20 mutational signatures	^a^MAF ≥ 0.02% (all gnomAD v.2.1.1.non-cancer populations) *OR* ≥ 10 homozygotes (source gnomAD non-cancer individuals)

*Abbreviations*: *CMMRD* constitutional mismatch repair deficiency, *gnomAD* Genome Aggregation Database (https://gnomad.broadinstitute.org/), *MAF* minor allele frequency, *MMR* DNA mismatch repair, *PPAP* polymerase proofreading-associated polyposis

^a^The recurrent germline *POLE* ED pathogenic variant p.Leu424Val has a MAF in gnomAD of 0%, and the maximum number of gnomAD individuals harboring a known ED pathogenic variant is 1 (1 in ~ 230,000 alleles for *POLD1* p.Asp316His).The established threshold in favor of neutrality implies the presence of the variant in ≥ 46 in 230,000 alleles

^b^PPAP tumors include adenomatous polyposis, CRC, endometrial cancer, breast cancer, ovarian cancer, extracolonic GI cancer or brain cancer

**Table 2:** Germline *POLE* and *POLD1* ED variants reported in the literature with strong evidence to be considered (likely) pathogenic or (likely) benign. The criteria considered for their selection as pathogenic or benign (criteria in Table 1) are highlighted in bold. Details and references are shown in Table S3

Variant	MAF^a^	Exo motif / Exonuclease catalytic site / DNA binding^b^	REVEL^c^	Somatic hotspot^d^ or recurrent in PPAP^e^	CMMRD-like phenotype	No. tumors with SBS10, 14, &/or 20 vs. total no. tumors^f^
**Pathogenic**						
*POLE*:c.824A>T; p.Asp275Val	**0**	Exo I-**catalytic** **(BLOSUM62: -3)**	0.817	No	No	n.a
*POLE*:c.830A>G; p.Glu277Gly	**0**	Exo I-**catalytic** **(BLOSUM62: -2)** < 6 Å from DNA	0.835	No	**CMMRD-like**	n.a
*POLE*:c.833C>A; p.Thr278Lys	**0**	Exo I < 6 Å from DNA	0.666	No	No	**6/6**
*POLE*:c.857C>G; p.Pro286Arg	**0**	Flanking Exo I < 6 Å from DNA	0.837	**Somatic hotspot** (*n* = 86 tumors)	No	**28/28**
*POLE*:c.881T>G; p.Met294Arg	**0**	Flanking Exo I < 6 Å from DNA	0.815	**Recurrent in PPAP** (*n* = 3 families)	No	**2/3**
*POLE*:c.890C>T; p.Ser297Phe	**0**	Outside Exo	0.799	**Somatic hotspot** (*n* = 17 tumors)	**CMMRD-like** ≥ 1 de novo	**5/5**
*POLE*:c.1089C>G; p.Asn363Lys	**0**	Exo II < 6 Å from DNA	0.735	**Recurrent in PPAP** (*n* = 3 families) ≥ 1 de novo	No	1/1
*POLE*:c.1231G>C; p.Val411Leu	**0**	Flanking Exo IV	0.457	**Somatic hotspot** (*n* = 73 tumors)	**CMMRD-like** ≥ 1 de novo	**23/23**
*POLE*:c.1270C>G; p.Leu424Val	**0**	Exo IV < 6 Å from DNA	0.654	**Recurrent in PPAP** (*n* = 24 families) ≥ 1 de novo	No	**7/7**
*POLE*:c.1307C>G; p.Pro436Arg	**0**	Exo V	0.592	No	**CMMRD-like**	**2/2**
*POLE*:c.1331T>A p.Met444Lys	**0**	Flanking Exo V < 6 Å from DNA	0.621	No	**CMMRD-like**	**2/2**
*POLE*:c.1366G >C; p.Ala456Pro	**0**	Exo III	0.620	**Somatic hotspot** (*n* = 19 tumors)	**CMMRD-like**	**5/5**
*POLE*:c.1381T>A; p.Ser461Thr	**0**	Exo III	0.587	No	**CMMRD-like** ≥ 1 de novo	**2/2**
*POLD1*:c.947A>G; p.Asp316Gly	**0**	Exo I-**catalytic** **(BLOSUM62: -1)** < 6 Å from DNA	0.773	No	No	0/1 (somatic dMMR)
*POLD1*:c.946G>C; p.Asp316His	**1/231076 (0.0004%)**	Exo I-**catalytic** **(BLOSUM62: -1)** < 6 Å from DNA	0.743	No	No	1/1 and *POLD1* cnLOH (germline, pMMR)
*POLD1*:c.1421T >C; p.Leu474Pro	**0**	Exo IV < 6 Å from DNA	0.913	**Recurrent in PPAP** (*n* = 5 families)	No	1/1 and *POLD1* cnLOH (germline, pMMR)
*POLD1*:c.1433G>A; p.Ser478Asn	**0**	Exo IV	0.377	**Recurrent in PPAP** (*n* = 6 families)	No	1/1 (somatic, dMMR) 7/7 (germline, pMMR) 1/1 and *POLD1* cnLOH (germline, pMMR)
**Benign**						
*POLE*:c.861T >A; p.Asp287Glu	**216/268316 (0.08%)**	Flanking Exo I	0.286	No (families with no PPAP phenotype)	No	0/2
*POLE*:c.1007A>G; p.Asn336Ser	**702/263956 (0.26%); 11 homoz**	outside	0.425	No	No	n.a
*POLE*:c.1145G>A; p.Ser382Asn	33/236892 (0.014%); 2 homoz **(0.1% in Asians)**	outside	0.055	No (only present in gnomAD individuals)	No	n.a
*POLD1*:c.1504G>A; p.Asp502Asn	**52/256992 (0.020%)**	Flanking Exo III	0.132	No (only present in gnomAD individuals)	No	n.a
*POLD1*:c.1562G>A; p.Arg521Gln	31/267602 (0.012%) **(0.024% in NFE)**	outside	0.278	No	No	0/1

*Abbreviations*: *CMMRD* constitutional mismatch repair deficiency, *dMMR* MMR deficiency, *homoz* homozygotes, *MAF* minor allele frequency, *MMR* mismatch repair, *n.a.* not available information, *NFE* non-Finnish Europeans, *pMMR* MMR proficiency, *PPAP* polymerase proofreading-associated polyposis

^a^Population MAF: GnomAD v.2.1.1, non-cancer individuals. MAF = 0 was considered when the variant was not reported in gnomAD but was in a region covered by the sequencing data (> 30X coverage) [59]

^b^Exo Motifs (I – V): POLE: Exo I, amino acids (aa.) 271–285; Exo II, aa. 359–372; Exo III, aa. 453–466; Exo IV, aa. 420–428; Exo V, aa. 430–438. POLD1: Exo I, aa. 312–326; Exo II, aa. 393–406; Exo III, aa. 506–519; Exo IV, aa. 470–478; Exo V, aa. 485–493). Exonuclease catalytic sites: POLE D275 and E277, and POLD1 D316 and E318. For DNA binding information, see Material and Methods for details and definitions, and Table S2 for specific values (predictions)

^c^REVEL score: 0–1; the closer to one, the higher pathogenicity prediction

^d^A variant was considered a somatic hotspot when present in ≥ 10 tumors (TCGA and COSMIC data considered; Table S5)

^e^A germline variant was considered recurrent in PPAP families when present in ≥ 3 PPAP-affected families

^f^Tumors from TCGA and COSMIC with available exome sequencing data, and tumors from hereditary cases with available mutational signature information reported in the literature, were considered

### *POLE* and *POLD1* ED-specific variant curation criteria

*POLE* and *POLD1* specifications to the ACMG/AMP criteria are shown in Table 3. Of the 28 original criteria, 8 were excluded (PVS1, PM3, PM4, PP2, PP5, BP1, BP3, and BP6). Rules were modified by detailing the content and/or changing the strength level of the original recommendations.

**Table 3:**
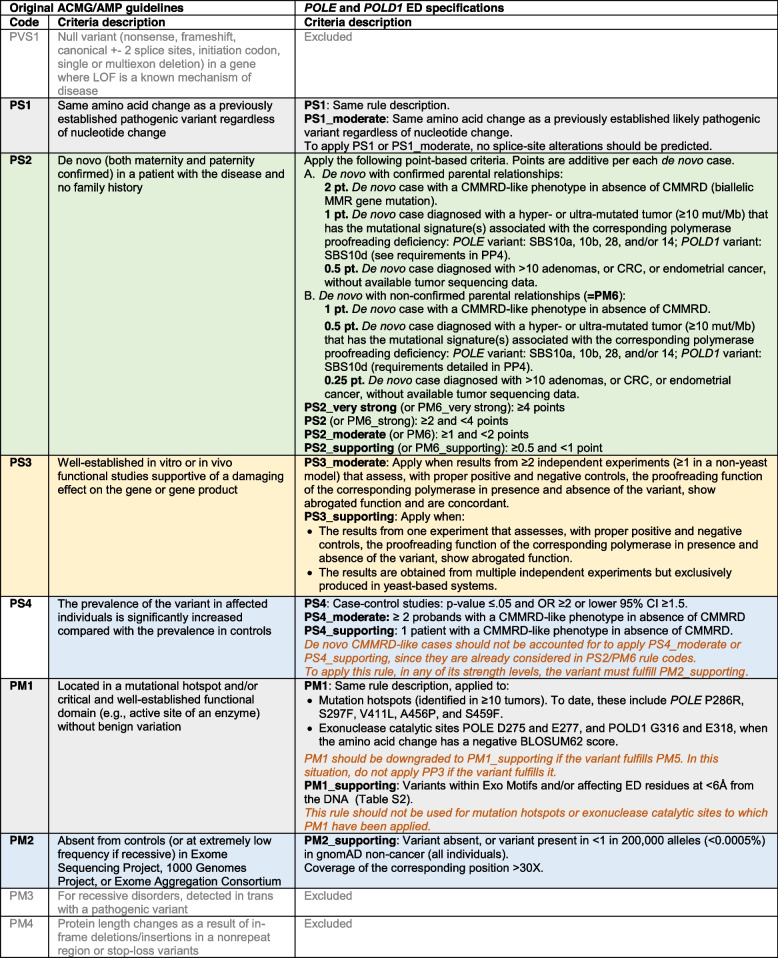

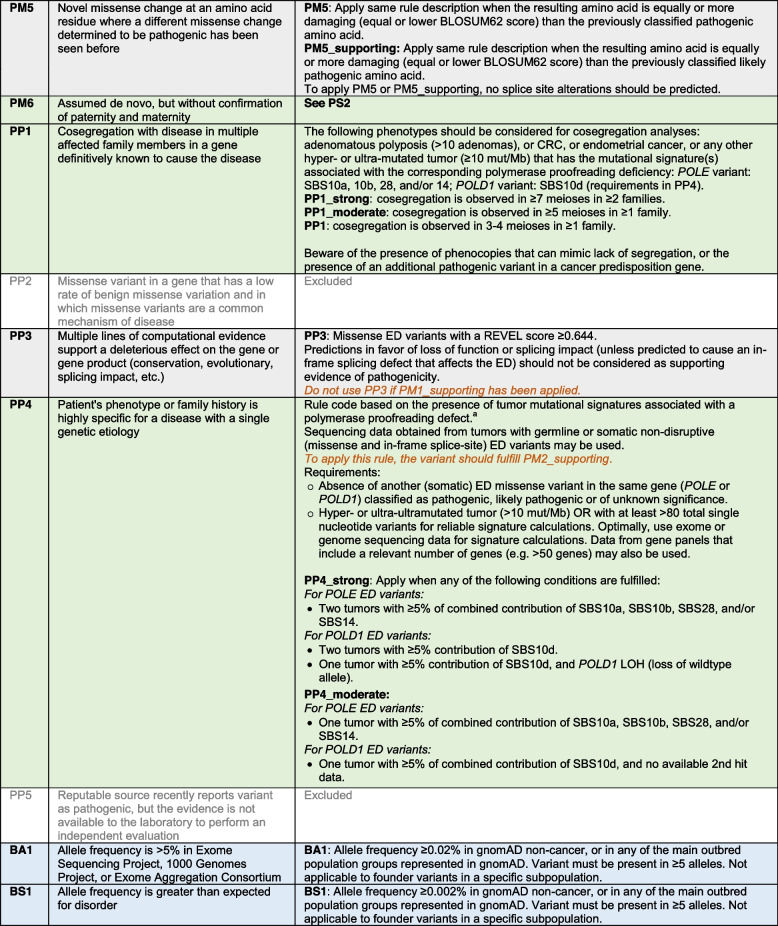

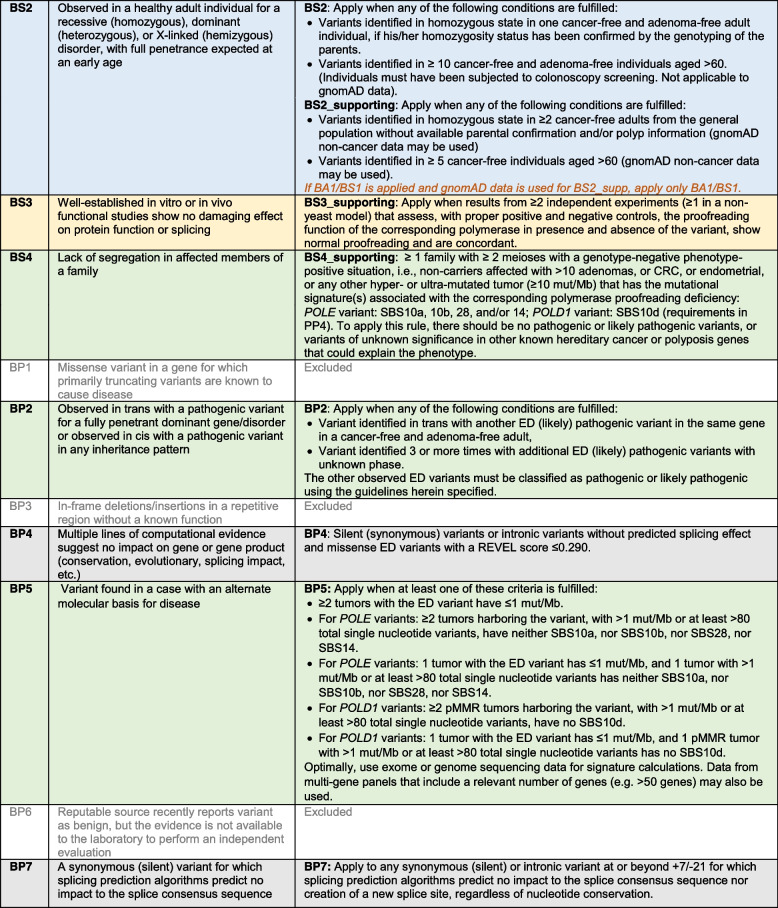
*POLE* and *POLD1* ED-specific ACMG/AMP recommendations. In blue, population data; in green, segregation and phenotypic data; in grey, variant nature, location and in silico predictive data; and in yellow, functional data. In orange italics, criteria combinations that are not allowed or criteria that need modification when co-used with another one

Tumor mutational signature analysis may be performed with any informatic tool available. The herein proposed recommendations have been tested with the results obtained with the web-based tool Signal (https://signal.mutationalsignatures.com/): For *POLE* variants the COSMIC v.3 signatures included in Signal were analyzed, which include, among others, SBS10a, 10b, 28, 14 and 20; for *POLD1* variants the Cancer Reference Signatures (CRS) included in Signal were analyzed, which include, among others, SBS10a, 10d, 14 and 20. In any case, no selection of the cancer type should be performed

#### Population data

*BA1* and *BS1* are criteria against pathogenicity based on the frequency of the variant in general population. To calculate the allele frequency threshold, the prevalence and penetrance of germline pathogenic variants in *POLE* and *POLD1* ED should be considered. Available data indicate that PPAP is a rare syndrome with very low population prevalence: Only one of 17 *POLE* ED pathogenic variants considered (Table 2) was detected in gnomAD non-cancer individuals (*POLD1:*c.946G>C; p.Asp316His: 1 in ~ 230,000 alleles). Although accurate unbiased penetrance estimates are still unavailable, available data [5, 6] suggests that the penetrance for *POLE* and *POLD1* ED pathogenic variants might be close to other autosomal dominant cancer syndromes caused by DNA repair defects, such as Lynch syndrome (*MLH1*, *MSH2*), with an estimated average CRC risk of ~ 40%—50% by age 70 [60]. By using the Whiffin/Ware calculator [61] (http://cardiodb.org/allelefrequencyapp/), the inferred allele frequency threshold (AFT) (95% CI) obtained for BA1, with allele heterogeneity set at 1, was 0.002%, and for BS1, with allele heterogeneity set at 0.1, 0.0002% (Additional file 1: Supplementary Results). Due to the scarcity of available data, the rough estimation of the syndrome penetrance, and the fact that the number of pathogenic variants is likely underestimated (missense variants are harder to classify than loss-of-function variants), we recommend applying higher AFTs: BA1 to variants with a population allele frequency ≥ 0.02%, and BS1 to variants with a population allele frequency ≥ 0.002%. Data may be obtained from gnomAD (non-cancer), or from any outbred (non-founder) population groups in that repository (non-Finnish European, African/African American, Latino/admixed American, South Asian, or East Asian). The variant must be present in at least 5 alleles.

*PM2* uses absence in controls for autosomal dominant diseases. Based on the incomplete penetrance and/or late disease onset, we recommend using PM2 with a supporting level of strength for variants absent, or present in ≤ 1 in 200,000 alleles (≤ 0.0005%) in gnomAD non-cancer dataset (all individuals) (coverage of variant position > 30X) [59]. Supportive of this threshold is the fact that *POLE* p.Leu424Val, the most recurrent known pathogenic germline variant, is not present in non-cancer gnomAD individuals (~ 200,000 in gnomAD v.2.1.1 and v.3.1.1).

*BS2* uses the presence of the variant in healthy adult individuals when full penetrance is expected at an early age. We specified the code to account for the reduced penetrance and later age of PPAP onset. Also, heterozygotes identified among non-cancer individuals could have polyps that have not been detected or reported. Considering the families with the 17 pathogenic variants listed in Table 2, among the 169 carriers reported (*POLE n* = 128 and *POLD1 n* = 41), there are 47 cancer-free individuals. Of them, 12 carriers had no polyp information and/or had not undergone colonoscopy screening. Of the cancer-free carriers with polyp information (35/47), 97% (34/35) had polyps (any number). Of these, detailed information on polyp number was specified for 25 individuals: 60% of them (15/25) had been diagnosed with ≥ 10 polyps (median age at diagnosis: 35; age range: 15–53) (Additional file 4: Table S4). Based on the available data, and on the extremely low prevalence of PPAP-associated recurrent pathogenic variants, we recommend using BS2, with a supporting level of strength, for variants that have been identified in ≥ 5 cancer-free individuals aged > 60. If BA1/BS1 is applied and gnomAD data is used for BS2_supp, apply only BA1/BS1. A strong level of strength may be applied if the variant is identified in ≥ 10 cancer-free and adenoma-free individuals aged > 60. To apply this level of strength, the cancer-free individuals must have been subjected to colonoscopy screening (not applicable for gnomAD individuals).

No biallelic germline ED pathogenic variants have been identified in humans. It has been speculated that those could likely be embryonic lethal [3]. Interestingly, depending on the nature of the pathogenic variant, biallelic mutant mice may be viable [62, 63]. While *Pole*^*P286R/P286R*^ mice showed embryonic lethality, homozygotes for other ED pathogenic variants survived into adulthood but developed cancer very early in life. In that same line, *Pole*^*P286R/*+ ^mice develop more severe phenotypes than heterozygotes for other ED pathogenic variants, which may even be indistinguishable from wildtype animals, suggesting a more severe effect in humans than in mice [62–64]. *Pold1 *homozygous mutant mice die of cancer at extremely early ages [63]. Based on the mice findings, even in the hypothetical case that biallelic ED-mutated humans were identified (viable), we would expect extremely aggressive tumor phenotypes, probably with very early age of onset. Therefore, we propose to apply BS2 to variants identified in homozygous state in one cancer- and adenoma-free adult individual if his/her homozygosity status has been confirmed by genotyping the parents. BS2_supporting may be applied when two homozygous adult cases are identified without available parental confirmation and/or polyp information (e.g. gnomAD non-cancer dataset).

*PS4* is based on the statistically significant higher frequency of the variant in patients compared to controls. We recommend applying the case–control criterion, considering PPAP-associated phenotypes (Table 4), when the resulting p-value is ≤ 0.05 and OR ≥ 2 or the lower 95% CI is ≥ 1.5 [43]. Also, we recommend applying PS4, with supporting level of strength, when a CMMRD-like phenotype [65] in absence of germline biallelic MMR (likely) pathogenic variants or VUSs is identified in one proband, and with moderate level of strength, when the CMMRD-like phenotype is identified in ≥ 2 probands. No other PPAP-associated phenotypes are considered due to their non-specificity. See Table 3 for permitted co-usages.

**Table 4:** Clinical phenotypes of PPAP considering the 169 carriers (122 cancer-affected) reported in the literature with any of the 17 pathogenic variants listed in Table 2. Columns 2–4 consider individual cancers (if one person was diagnosed with several primary tumors, they are individually accounted for). Columns 5–7 consider the number of carriers with a specific phenotype, See Table S4 for details

Clinical phenotypes	Cancers in *POLE* carriers (%)	Cancers in *POLD1* carriers (%)	Cancers in *POLE* & *POLD1* carriers (%)	*POLE* carriers (%)	*POLD1* carriers (%)	*POLE* & *POLD1* carriers (%)
	***Cancers***^***g***^			**C*ancer-affected carriers***^***g***^		
**Total**	**164**	**48**	**212**	**94**	**28**	**122**
CRC	102 (62.20%)	27 (56.25%)	129 (60.85%)	76 (80.85%)	20 (71.43%)	96 (78.69%)
*Median age (range)*	*Age: 45 (13–88)*	*Age: 39.7 (21–80)*	*Age: 43.7 (13–88)*	*Age: 41 (13–88)*	*Age: 39.6 (21–80)*	*Age: 41.6 (13–88)*
Endometrial cancer^a^	11/87 (12.64%)	11/36 (30.56%)	22/123 (18.89%)	11/41(26.83%)	11/20 (55.00%)	22/61 (36.07%)
*Median age (range)*	*Age: 50 (30–56)*	*Age: 50 (31–58)*	*Age: 48.8 (30–58)*	*Age: 50 (30–56)*	*Age: 50 (31–58)*	*Age: 48.8 (30–58)*
Breast cancer^a^	7/87 (8.05%)	5/36 (13.89%)	12/123 (9.76%)	7/41 (17.07%)	4/20 (22.00%)	11/61 (18.03%)
*Median age (range)*	*Age: 50 (38–65)*	*Age: 62.6 (52–75)*	*Age: 52.3 (38–75)*	*Age: 50 (38–65)*	*Age: 59.5 (52–65)*	*Age: 53.5 (38–65)*
Ovarian cancer^a^	8/87 (9.20%)	0/36 (0%)	8/123 (6.50%)	7/41 (17.07%)	0/20 (0%)	7/61 (11.48%)
*Median age (range)*	*Age: 37 (33–50)*	n.a	*Age: 37 (33–50)*	*Age: 37 (33–50*)	n.a	*Age: 37 (33–50)*
Extracolonic GI cancer^b^	12 (7.32%)	1 (2.08%)	13 (6.13%)	12 (12.77%)	1 (3.57%)	13 (10.66%)
*Median age (range)*	*Age: 53.5 (35–78)*	*Age: 36 (36–36)*	*Age: 52 (35–78)*	*Age: 53.5 (35–78)*	*Age: 36 (36–36)*	*Age: 52 (35–78)*
Brain cancer	17 (10.37%)	2 (4.17%)	19 (8.96%)	17 (18.08%)	2 (7.14%)	18 (14.75%)
*Median age (range)*	*Age: 30 (4–66)*	*Age:* 26 (26–26)	*Age: 29 (4–66)*	*Age: 30 (4–66)*	*Age: 26 (26–26)*	*Age: 29.5 (4–66)*
Other cancers^c^	7 (4.27%)	2 (4.17%)	9 (4.24%)	7 (7.45%)	2 (7.14%)	9 (7.38%)
*Median age (range)*	*Age: 47 (31–71)*	*Age: 60.5 (56–65)*	*Age: 48 (31–71)*	*Age: 47 (31–71)*	*Age: 60.5 (56–65)*	*Age: 49.5 (31–71)*
Multiple primary cancers	-	-	-	28 (29.79%)	11 (39.3%)	39 (32%)
*Median age*				*Age: 46 (11–76)*	*Age: 44.7 (26–65)*	*Age: 45.6 (11–76)*
				***Carriers***		
**Total**				**128**	**41**	**169**
Individuals affected with neoplastic and preneoplastic lesions, and non-tumoral extracolonic manifestations^d^	-	-	-	98 (76.56%)	28 (68.29%)	126 (74.56%)
Median age (range)				*Age:42 (1–88)*	*Age: 45 (21–80)*	*Age: 42 (1–88)*
Cancer-free^e^	-	-	-	34 (26.56%)	13 (31.70%)	47 (27.81%)
Carriers with > 10 polyps reported^f^	-	-	-	41 (61.19%)	12 (50.00%)	53 (58.24%)
*Median age (range)*				*Age: 35 (13–67)*	*Age: 40 (28–53)*	*Age: 37 (13–67)*

^a^Only females considered (123 cancers; 87 *POLE* and 36 *POLD1*; 61 cancer-affected female carriers; 41 *POLE* and 20 *POLD1*)

^b^Extracolonic GI cancer: gastric cancer, pancreatic cancer, small intestine cancer, duodenal cancer, esophageal cancer, and gastrointestinal stromal tumors

^c^Other phenotypes: prostate cancer, kidney cancer, skin cancer, ureter cancer, neuroendocrine colon cancer, and mesothelioma

^d^Total calculated considering all phenotypes: cancers, benign/premalignant tumors (e.g. polyps), and non-tumoral extracolonic manifestations (e.g. café-au-lait macules)

^e^Age information for cancer-free individuals is very scarce in the literature and was not included

^f^Frequency calculated based on 91 carriers with polyp information (67 *POLE* and 24 *POLD1 *carriers)

^g^Total calculated considering cancer phenotypes. Polyps, benign tumors and other non-tumoral manifestations were not included

#### *Variant nature and location, and *in silico* predictions*

Evidence suggests that loss-of-function and outside-ED *POLE* and *POLD1 *variants are nonpathogenic for PPAP, and only missense and in-frame indel variants within the ED should be considered as potential cause of PPAP and as predictive biomarkers in oncology [3, 5, 66, 67]. Therefore, PVS1, PM4, BP1, and BP3 are not considered due to their irrelevance to the syndrome and its mechanism of pathogenicity.

The *PM1* criterion is given to mutational hotspots and/or critical well-established functional domains without benign variation. We recommend applying PM1 (moderate) for: (i) somatic mutational hotspots (observed in ≥ 10 tumors), which currently include: *POLE* P286R, S297F, V411L, A456P and S459F (somatic hotspot information obtained from TCGA and COSMIC tumors: Additional file 5: Table S5); and (ii) variants affecting the exonuclease catalytic sites *POLE* D275 and E277, and *POLD1 *D316 and E318 [4], when the resulting amino acid shows a negative BLOSUM62 when compared to the wildtype residue. Available data indicate that variants affecting the binding of the exonuclease with the DNA, and/or located within the Exo motifs are likely to be pathogenic [68]. In fact, 11 of the 13 non-catalytic pathogenic variants, and none of the benign variants, affect residues of Exo motifs and/or are in contact (distance < 6 Å) with the DNA when the polymerase is in proofreading position (Table 2). We recommend applying PM1 with a supporting level of strength to any variant fulfilling either one of these two conditions, when PM1 (moderate) has not been applied. ED amino acids at < 6 Å from the DNA are listed in Additional file 4: Table S2. See Table 3 for permitted co-usages of PM1 with other criteria.

*PP3* and *BP4 *are related to in silico pathogenicity predictions. Following recent ClinGen indications [69], *PP3* should be applied for variants with REVEL scores ≥ 0.644, which occurs for 11 of the 17 pathogenic variants and none of the benign variants, and *BP4* for silent (synonymous) variants or intronic variants without predicted splicing effect, and for missense ED variants with a REVEL score ≤ 0.290. Sensitivity/specificity analysis should be performed to set gene-specific cutoff values for *POLE* and *POLD1* ED variants, when enough pathogenic and benign variants are identified to be used for the analysis. Predictions of loss of function or splicing impact (unless it causes an in-frame splicing defect that affects the ED) should not be considered as supporting evidence of pathogenicity or benignity.

*BP7* is applied for any synonymous or intronic variant at or beyond +7/-21 for which splicing prediction algorithms predict no impact to the splice consensus sequence nor creation of a new splice site, regardless of nucleotide conservation.

*PS1* considers any missense nucleotide change that translates into an amino acid change that has been previously established as (likely) pathogenic with a different nucleotide change (i.e., different nucleotide variant, same amino acid change). Strong level of evidence is recommended for pathogenic variants, and moderate, for likely pathogenic variants. Likewise, *PM5* relates to a missense variant at a residue where a different pathogenic missense variant caused a change to a different amino acid. In this case, we recommend using PM5 only when the resulting amino acid shows equal or lower BLOSUM62 score (i.e., equally or more damaging) than the previously classified pathogenic (*PM5*) or likely pathogenic (PM5_supporting) amino acid [70].

#### Segregation and phenotypic data

*PP1* original criterion uses cosegregation of the variant with the disease in multiple family members affected with the associated phenotype as evidence for pathogenicity. The main PPAP-associated tumor types are colorectal, endometrial, ovarian, breast, brain, and upper gastrointestinal cancers, as well as polyposis (> 10 adenomas), all with prevalence values > 10% among cancer-affected carriers (Table 4; Additional file 4: Table S4). Nevertheless, due to the broad phenotypic spectrum and the relative high population frequency of most PPAP-associated tumor types, which may lead to phenocopies, we recommend considering only the three most prevalent PPAP-associated phenotypes, i.e. adenomatous polyposis (> 10 adenomas), CRC and endometrial cancer, unless tumor mutational data indicate that other tumor types are hyper/ultra-mutated and harbor the gene-specific mutational signature(s).

Based on the gradations considered by ClinGen variant curation expert panels [39, 40, 42, 71], we recommend the system that considers the number of meiosis across one or more families [72]: strong level of evidence when co-segregation is observed in ≥ 7 meiosis in ≥ 2 families; moderate level of evidence when cosegregation is observed in ≥ 5 meioses in ≥ 1 family; and supporting level when cosegregation is observed in 3–4 meioses in ≥ 1 family. The meiosis counting-based system may not be optimal for cosegregation analyses in cancer-related genes [72], particularly when there are variable ages at onset, high probability of phenocopies, and/or incomplete penetrance, as happens for PPAP. When more accurate data on the syndrome are available, this rule code will likely implement a Bayes factor-based approach, which measures the likelihood that cosegregation patterns represent a gene-disease penetrance model [72].

*BS4* is used when there is lack of segregation. Due to existence of de novo cases, the wide tumor spectrum observed in PPAP, the expected incomplete penetrance and the -often- late onset of cancer, we recommend considering only non-carrier family members affected with > 10 adenomas, or CRC, or endometrial, or any other hyper- or ultra-mutated tumor (≥ 10 mut/Mb) with the mutational signature(s) associated with the corresponding polymerase proofreading deficiency. BS4 should be applied, with a supporting level of strength, when there is ≥ 1 family with ≥ 2 meiosis with a genotype-negative phenotype-positive situation, in absence of pathogenic or likely pathogenic variants or variants of unknown significance in other known hereditary cancer or polyposis genes that could explain the phenotype. As for PP1, this criterion will likely implement a Bayes factor–based approach [72] in the future.

*PS2* and *PM6* contemplate the presence of de novo variants. We recommend applying the point-based criteria based on phenotypes indicated in Table 3 to determine the levels of strength. Points are additive per each de novo case.

We recommend applying *BP2* when the variant is observed *in trans* with another (likely) pathogenic ED variant in the same gene in a tumor-free (cancer- and adenoma-free) adult (see comment in “Population data” section; BS2 criterion) or when the variant is identified ≥ 3 times with additional ED (likely) pathogenic variants in the same gene with unknown phase. The other observed ED variant must have been classified as (likely) pathogenic using the herein defined recommendations.

#### Tumor data: mutational burden and signatures

**Fig. 1:**
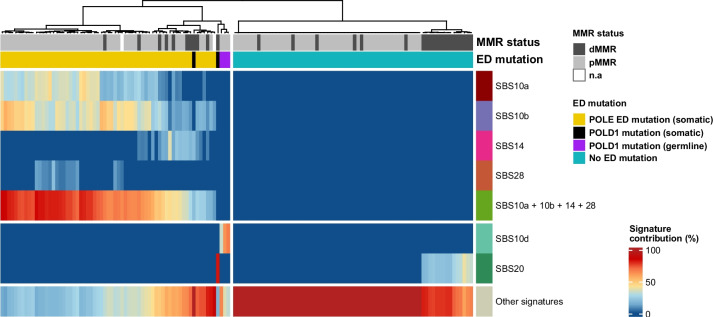
Heatmap showing the clustering of tumors based on the contribution of tumor mutational signatures SBS10a, SBS10b, SBS10d, SBS28, SBS14, SBS20 and “other signatures”. Analysis was performed with 64 tumor samples with somatic pathogenic variants in *POLE* and *POLD1* EDs, 3 tumors belonging to three probands with germline pathogenic variants in *POLD1*, and 70 TCGA tumor samples without polymerase exonuclease domain variants

To evaluate the specificity of the proofreading-associated mutational signatures, we analyzed 134 tumor samples (different tumor types) including: i) 50 MMR proficient (pMMR) and 20 dMMR TCGA tumors without ED variants, and ii) 50 pMMR, 12 dMMR tumors and 2 tumors without available MMR status information with somatic pathogenic ED variants (62 tumors with *POLE* and 2 with *POLD1* ED mutations) that represent 9 of the 17 pathogenic variants listed in Table 2 (data source: 59 TCGA tumors and 5 COSMIC tumors with available exome sequencing data). The results are represented as a Heatmap in Fig. 1 (details in Additional file 6: Table S6). SBS10a, SBS10b, SBS28 and SBS14 were highly specific of Polε proofreading deficiency; no trace of those signatures was detected among the tumors without ED variants. SBS14 was mostly, although not exclusively, found among dMMR tumors.

Only two *POLD1* ED-mutated tumors, both dMMR, could be included in the analysis: one tumor had 10% SBS14 contribution and no trace of Polδ proofreading-deficient signatures (SBS10d or SBS20), and the other had 83% SBS20 contribution. Unlike the other polymerase proofreading-associated signatures, SBS20 was also observed in a subgroup of dMMR tumors (*n* = 15) without ED variants, at contributions ranging from 18 to 40%. Due to its non-specificity, we recommend not using SBS20 for variant classification. Due to the lack of pMMR, Polδ proofreading-deficient sporadic tumors, we re-analyzed exome/genome sequencing data obtained from three additional proofreading-deficient tumors (two CRCs and one adenoma), developed by heterozygous carriers of germline *POLD1 *p.Leu474Pro, p.Asp316His, and p.Ser478Asn [54, 73]. All three samples were hypermutated (59, 114 and 36 mut/Mb respectively) and had 34%-68% contribution of SBS10d, highly specific of Polδ proofreading deficiency in tumors (Fig. 1). Moreover, all three tumors had copy-neutral loss of heterozygosity (cnLOH) in the *POLD1 *region that caused the loss of the wildtype allele [73].

The 50 pMMR Polε proofreading-deficient cancers had an average of 144 mut/Mb (range: 2.6—325), and the 10 dMMR Polε proofreading-deficient cancers, 255 mut/Mb (range: 109 – 531). Only 2 samples, both harboring *POLE* p.Leu424Val had TMBs < 25 mut/Mb (2.6 and 4.4 mut/Mb). All 62 *POLE* ED-mutated tumors, regardless of their MMR status, had > 5% contribution of signatures SBS10a and/or 10b (median: 65%; range: 6%– 87%). When considering all Polε proofreading-deficient signatures combined, i.e. SBS10a, SBS10b, SBS28 and SBS14, 100% of samples reached > 20% contribution.

In the generic ACMG/AMP guidelines, PP4 corresponds to highly specific phenotypes or family history of a disease with a single genetic etiology, and BP5, to variants found in cases with an alternate molecular disease basis. We propose to adapt these criteria to the presence or absence of the proofreading deficiency-specific mutational signatures and high TMB. To consider PP4, no other (somatic) ED missense variant classified as (likely) pathogenic or of unknown significance in the same gene (*POLE* or *POLD1*) should occur in the tumor, and at least PM2_supporting must be fulfilled. We recommend performing the mutational signature analysis when the tumors are hypermutated (> 10 mut/Mb) or have at least a total of 80 somatic SNVs, to minimize the detection of false (artifact) signatures generated from an extremely small number of variants. Optimally, the use of exome or genome sequencing data is recommended, although the use of sequencing data obtained from panels that include a relevant number of genes may also be used.

We recommend using *PP4* with a strong level of strength: For *POLE* ED variants, when at least two tumors have SBS10a, SBS10b, SBS28, and/or SBS14; and for *POLD1* ED variants, when at least two tumors have SBS10d or when one tumor has SBS10d and loss of heterozygosity (LOH) that causes the loss of the wildtype allele. PP4_moderate may be applied for *POLE* ED variants when one tumor has SBS10a, SBS10b, SBS28, and/or SBS14; and for *POLD1* ED variants when there is one tumor with SBS10d (no available 2^nd^ hit information or no LOH). These recommendations are based on the data obtained from fresh/frozen tumor samples. To minimize the potential effect of FFPE sequencing artifacts, a ≥ 5% contribution of the gene-specific signatures will be considered to apply PP4 strong and moderate criteria.

We recommend using *BP5* when two or more tumors with the ED variant have ≤ 1 mut/Mb. For *POLE* variants, BP5 should be used when two or more tumors harboring the variant, with > 1 mut/Mb or at least > 80 total single nucleotide variants, have neither SBS10a, nor SBS10b, nor SBS28, nor SBS14; or when one tumor has ≤ 1 mut/Mb and another one, with > 1 mut/Mb or > 80 single nucleotide variants, has neither SBS10a, nor SBS10b, nor SBS28, nor SBS14. For *POLD1* variants, use BP5 when two or more pMMR tumors harboring the variant, with > 1 mut/Mb or at least > 80 total single nucleotide variants, do not have SBS10d; or when one tumor has ≤ 1 mut/Mb and one pMMR tumor, with > 1 mut/Mb or at least > 80 total single nucleotide variants, has no SBS10d. In all instances, at least two tumors are required to minimize the possible analysis of phenocopies and the effect of FFPE-derived sequencing artifacts.

#### Functional data

Available in vitro assays to test the functionality of *POLE* and *POLD1* ED variants assess the proofreading ability of the polymerases in absence and presence of the variant. The studies reported to date rely mostly on yeast-based assays, although cell-free assays, in vitro human or murine cell line experiments, and in vivo mouse models, have also been used (Additional file 3: Table S3).

*PS3* and *BS3* rely on well-established in vitro or in vivo functional studies supporting or discarding a damaging effect of the variant. Based on available data and the fact that the performance of the functional studies published so far has not been evaluated, we recommend using PS3_moderate when results from at least 2 independent experiments (at least one in a non-yeast model) that assess, with proper positive and negative controls, the proofreading function of the corresponding polymerase in presence and absence of the variant, show defects and are concordant. If only results from one experiment are available, or the results, even from multiple experiments, are produced exclusively in yeast-based systems [5], we recommend applying a supporting level of strength. We propose to decrease the level of strength for yeast-based evidence because published results show high variability among replicates and experiments (publications in Additional file 3: Table S3), and some concerns have been raised regarding the assessment of variants affecting the DNA binding, which might show an effect in yeast even when the variant is non-pathogenic [5, 10, 68]. We currently recommend using BS3_supporting, when at least two independent experiments (≥ 1 in a non-yeast model) show no proofreading defect. For both PS3 and BS3 criteria, the assayed amino acid change must be the same as the one identified in the patient.

The ClinGen Sequence Variant Interpretation Committee recommends assessing the performance of any functional assay using variants classified as pathogenic or benign according to clinical parameters (cross validation) [74], which has not been done for any of the *POLE/POLD1* functional assays reported to date. Calibration according to the cross-validation results is recommended to correctly apply the PS3 and BS3 rules, providing the correct level of strength, or a calibrated quantitative value if Bayesian transformation of the ED-specific ACMG/AMP guidelines is applied.

### Classification of reported variants

The defined classification recommendations (Table 3) were applied to 128 variants reported in the literature (reviewed: March 2023) and ClinVar (access date: July 2021), including the 23 variants used for the definition of the guidelines. Of the 128 variants considered, 7 were classified as pathogenic, 10 as likely pathogenic, 7 as benign, and 10 as likely benign. Of the 17 a priori pathogenic variants included in Table 2, all but *POLE*:c.824A>T; p.(Asp275Val) and *POLE*:c.830A>G; p.(Glu277Gly), now classified as variants of unknown significance, were classified as P (*n* = 7) or LP (*n* = 8). Moreover, two additional variants were classified as likely pathogenic: *POLE*:c.857C>T; p.(Pro286Leu) and *POLE*:c.1373A>T; p.(Tyr458Phe). Additional file 3: Table S3 shows the classification of all 128 variants taking into consideration the data available.

### Clinical features of reported individuals with constitutional *POLE* or *POLD1* ED pathogenic or likely pathogenic variants

To date, literature reports include 205 individuals heterozygous for the 17 *POLE* or *POLD1* variants classified as pathogenic or likely pathogenic following the defined recommendations. Of the 205 heterozygotes, 149 (73%) were diagnosed with cancer: 120 (58% of the 205 carriers) with CRC (mean age at diagnosis: 41; range: 13–80), 21 (22% of 95 female carriers) with endometrial cancer (age: 50; range: 31–58), 11 (12% of female carriers) with breast cancer (age: 55; range: 38–65); 8 (8% of female carriers) with ovarian cancer (age: 42; range: 33–50), 19 (9%) with extracolonic gastrointestinal cancers (age: 45; range: 35–78), 18 (9%) with brain cancer (age: 28; range: 4–66), and 9 (4%) with other cancer types. The majority of heterozygotes (88%) had reports of cancer, and/or preneoplastic lesions, and/or non-tumoral extracolonic manifestations (e.g. café-au-lait macules). Sixty-four percent of those with polyp information (70/108) were reported to have > 10 gastrointestinal polyps (detailed phenotypes in Additional file 4: Table S4).

While these phenotypes should currently guide clinical surveillance in carriers, future prospective collaborative efforts will provide more accurate (unbiased) estimates of cancer risk and penetrance. Furthermore, oncologic therapeutic decisions in the context of the hereditary cancer syndrome, and for cancers with somatic pathogenic or likely pathogenic *POLE* or *POLD1 *exonuclease variants, should consider the good prognosis and response to immune checkpoint inhibitors of polymerase proofreading deficient tumors [75–77].

## Conclusions

**Fig. 2:**
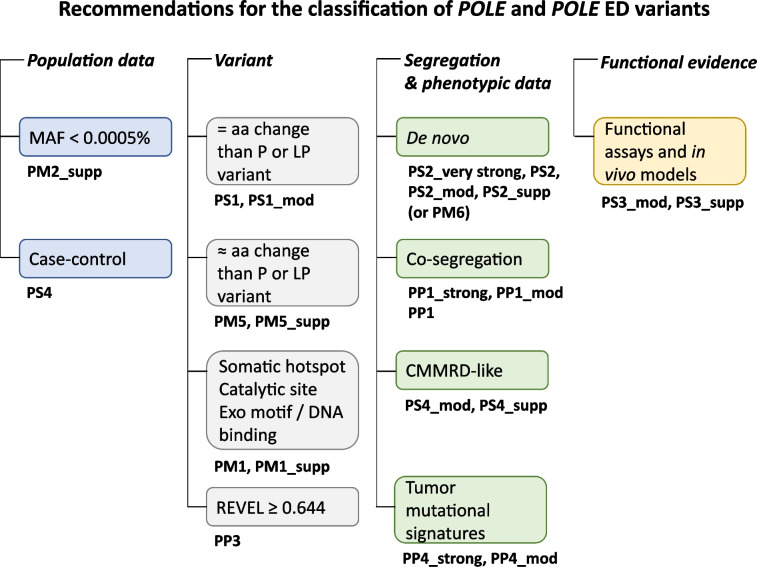
Schematic summary of the evidence that supports pathogenicity of ED variants

We propose the first recommendations based on the general ACMG/AMP guidelines for the classification of variants in the exonuclease domain of *POLE* and *POLD1*, taking into consideration the available evidence (Table 3, Fig. 2). With better phenotypic and molecular characterization of the syndrome and associated tumors, together with access to better and cross validated functional assays, improved recommendations are expected in following years.

### Supplementary Information

**Additional file 1: Supplementary Results.** Population allele frequency threshold (AFT) calculation. **Table S1.** Standard ACMG/AMP combination rules to define pathogenic, likely pathogenic, likely benign and benign variants.

**Additional file 2: Table S2.** Location of POLE and POLD1 amino acids in the 3D structure and their accessibility to the DNA.

**Additional file 3: Table S3.** Characteristics and classification of constitutional (germline) *POLE* and *POLD1* exonuclease domain missense variants.

**Additional file 4: Table S4.** Phenotypic features of reported individuals with the *POLE* and *POLD1* ED germline pathogenic variants listed in Table 2 and of heterozygous carriers of two additional variants reclassified as pathogenic or likely pathogenic after the application of the recommendations defined in this article.

**Additional file 5: Table S5.** TCGA and COSMIC tumors with the *POLE* or *POLD1* ED variants evaluated in this study.

**Additional file 6: Table S6.** Tumors evaluated in this study with available sequencing data for the calculation of tumor mutational burden and mutational signatures.

## Data Availability

Data supporting the reported results may be found in the article, supplementary material, public repositories (TCGA, COSMIC, gnomAD, ClinVar) and/or published articles.
