# Supplementary material for: Recommendations for the classification of germline variants in the exonuclease domain of POLE and POLD1
Source: Genome Med. 2023 Oct 17;15:85. doi: 10.1186/s13073-023-01234-y (PMC10580551; doi:10.1186/s13073-023-01234-y)
Supplement: Supplementary file 1 — Additional file 1: Supplementary Results. Population allele frequency threshold (AFT) calculation. Table S1. Standard ACMG/AMP combination rules to define pathogenic, likely pathogenic, likely benign and benign variants. [file 13073_2023_1234_MOESM1_ESM.pdf]

ADDITIONAL FILE 1

Supplementary Results

Population allele frequency threshold (AFT) calculation

To obtain a *POLE* and *POLD1* specific AFT we used the Whiffin/Ware calculator[1] (http://cardiodb.org/allelefrequencyapp/). We applied a conservative penetrance estimate (on the lower range) of 30%, and a prevalence of familial CRC of 1 in 770 in the general population (calculated from the Swedish nationwide cohort data: 12,829,251 people, 16,679 of whom are CRC patients with 1st or 2nd-degree relatives affected with CRC).[2] Data obtained by our group showed that the prevalence of *POLE* and *POLD1* pathogenic variants among familial CRC patients is ~0.6% (5/795 nonrelated CRC-affected individuals)[3-5]. Other relatively large studies[6-9] also detected prevalence values for *POLE* and *POLD1* pathogenic variants among familial CRC patients below 1%, only one[10] slightly exceeding that figure (~1.1%) (calculations made considering the pathogenic variants listed in Table 2). Based on these data, a value of 1% for the genetic contribution of *POLE* and *POLD1* pathogenic variants was considered (genetic heterogeneity: 0.01). Taking into consideration a 95% confidence interval (CI), the inferred AFT obtained for BA1, with allele heterogeneity set at 1, was 2.16×10^{-5} (0.002%) and for BS1, with allele heterogeneity set at 0.1, 2.16×10^{-6} (0.0002%).

Table S1. Standard ACMG/AMP combination rules to define pathogenic, likely pathogenic, likely benign and benign variants.[11]

**Pathogenic**	**Likely pathogenic**	**Likely benign**	**Benign**
≥ 2 Strong	1 Strong AND 1-2 Moderate	1 Strong AND 1 Supporting	1 Stand Alone (BA)
1 Strong AND:<br>a. ≥ 3 Moderate OR<br>b. 2 Moderate AND ≥ 2 Supporting OR<br>c. 1 Moderate AND ≥ 4 Supporting	1 Strong AND ≥ 2 Supporting	≥ 2 Supporting	≥ 2 Strong
	≥ 3 Moderate		
	2 Moderate AND ≥ 2 Supporting		
	1 Moderate AND ≥ 4 Supporting		

REFERENCES

1. Whiffin N, Minikel E, Walsh R, et al. Using high-resolution variant frequencies to empower clinical genome interpretation. Genet Med. 2017;19(10):1151-1158.
2. Tian Y, Kharazmi E, Sundquist K, Sundquist J, Brenner H, Fallah M. Familial colorectal cancer risk in half siblings and siblings: nationwide cohort study. BMJ. 2019;364:l803.
3. Valle L, Hernández-Illán E, Bellido F, et al. New insights into POLE and POLD1 germline mutations in familial colorectal cancer and polyposis. Hum Mol Genet. 2014;23(13):3506-3512.
4. Bellido F, Pineda M, Aiza G, et al. POLE and POLD1 mutations in 529 kindred with familial colorectal cancer and/or polyposis: review of reported cases and recommendations for genetic testing and surveillance. Genet Med. 2016;18(4):325-332.
5. Mur P, García-Mulero S, Del Valle J, et al. Role of POLE and POLD1 in familial cancer. Genet Med. 2020.
6. Palles C, Cazier JB, Howarth KM, et al. Germline mutations affecting the proofreading domains of POLE and POLD1 predispose to colorectal adenomas and carcinomas. Nat Genet. 2013;45(2):136-144.
7. Elsayed FA, Kets CM, Ruano D, et al. Germline variants in POLE are associated with early onset mismatch repair deficient colorectal cancer. Eur J Hum Genet. 2015;23(8):1080-1084.
8. Buchanan DD, Stewart JR, Clendenning M, et al. Risk of colorectal cancer for carriers of a germ-line mutation in POLE or POLD1. Genet Med. 2018;20(8):890-895.
9. Palles C, Martin L, Domingo E, et al. The clinical features of polymerase proof-reading associated polyposis (PPAP) and recommendations for patient management. Fam Cancer. 2021.
10. Hamzaoui N, Alarcon F, Leulliot N, et al. Genetic, structural, and functional characterization of POLE polymerase proofreading variants allows cancer risk prediction. Genet Med. 2020.
11. Richards S, Aziz N, Bale S, et al. Standards and guidelines for the interpretation of sequence variants: a joint consensus recommendation of the American College of Medical Genetics and Genomics and the Association for Molecular Pathology. Genet Med. 2015;17(5):405-424.
